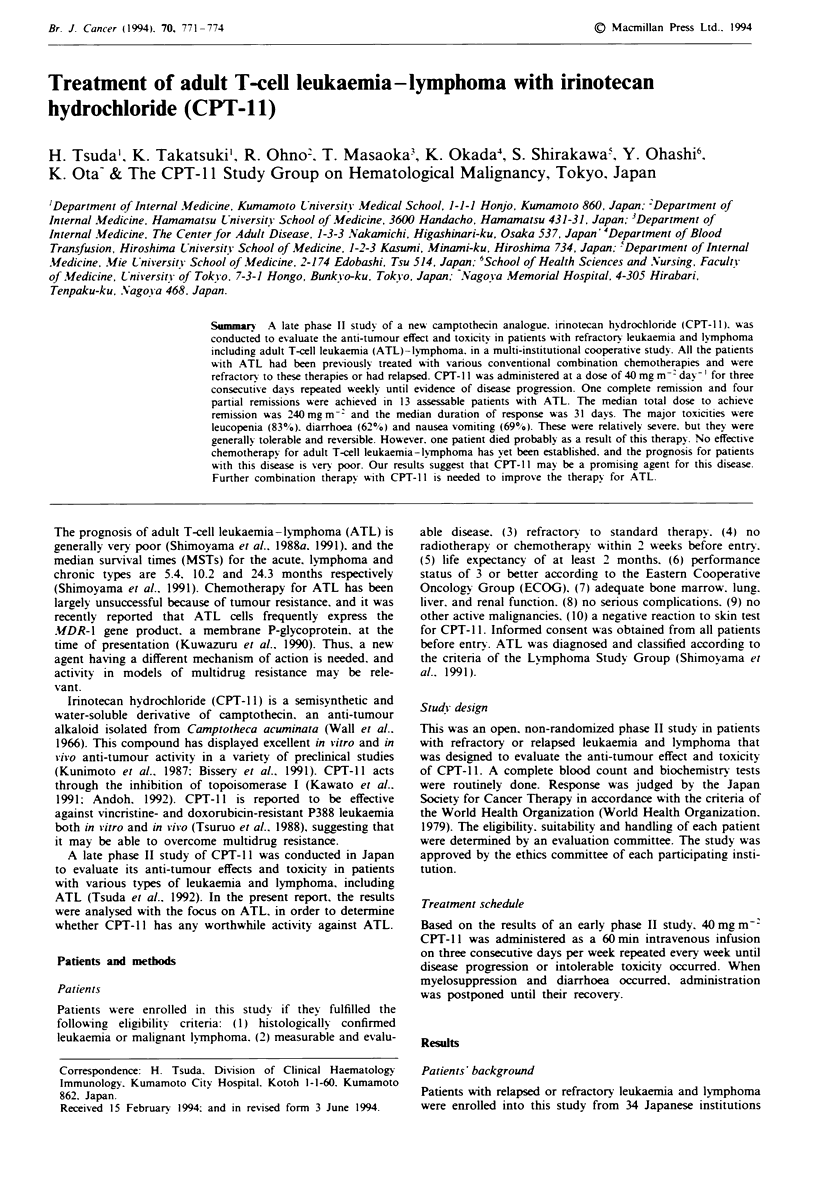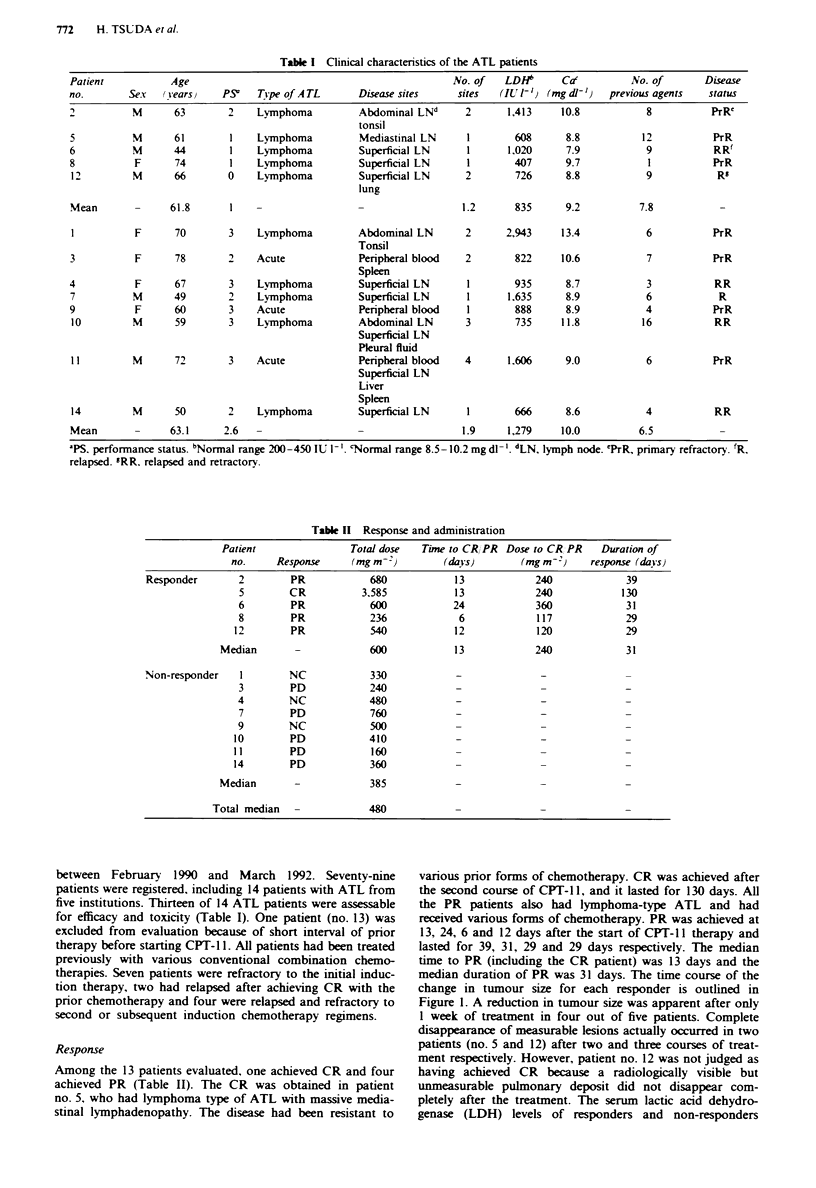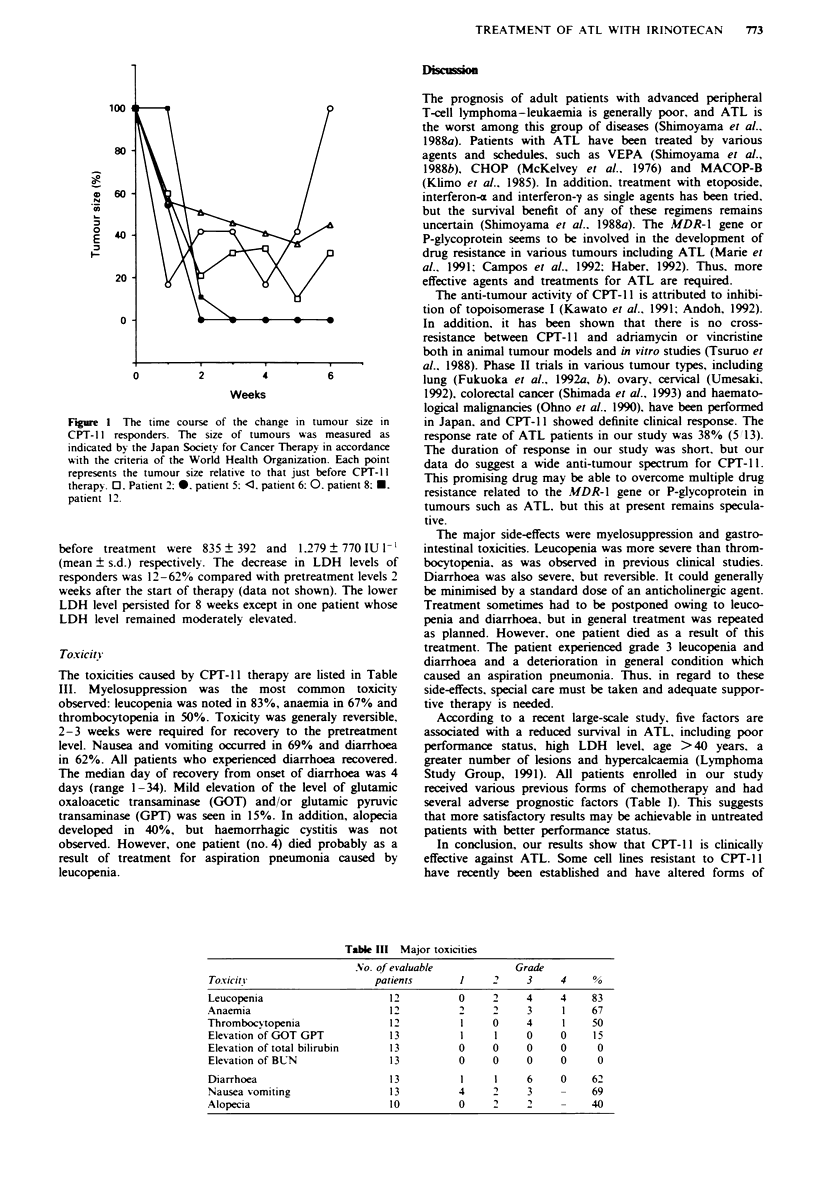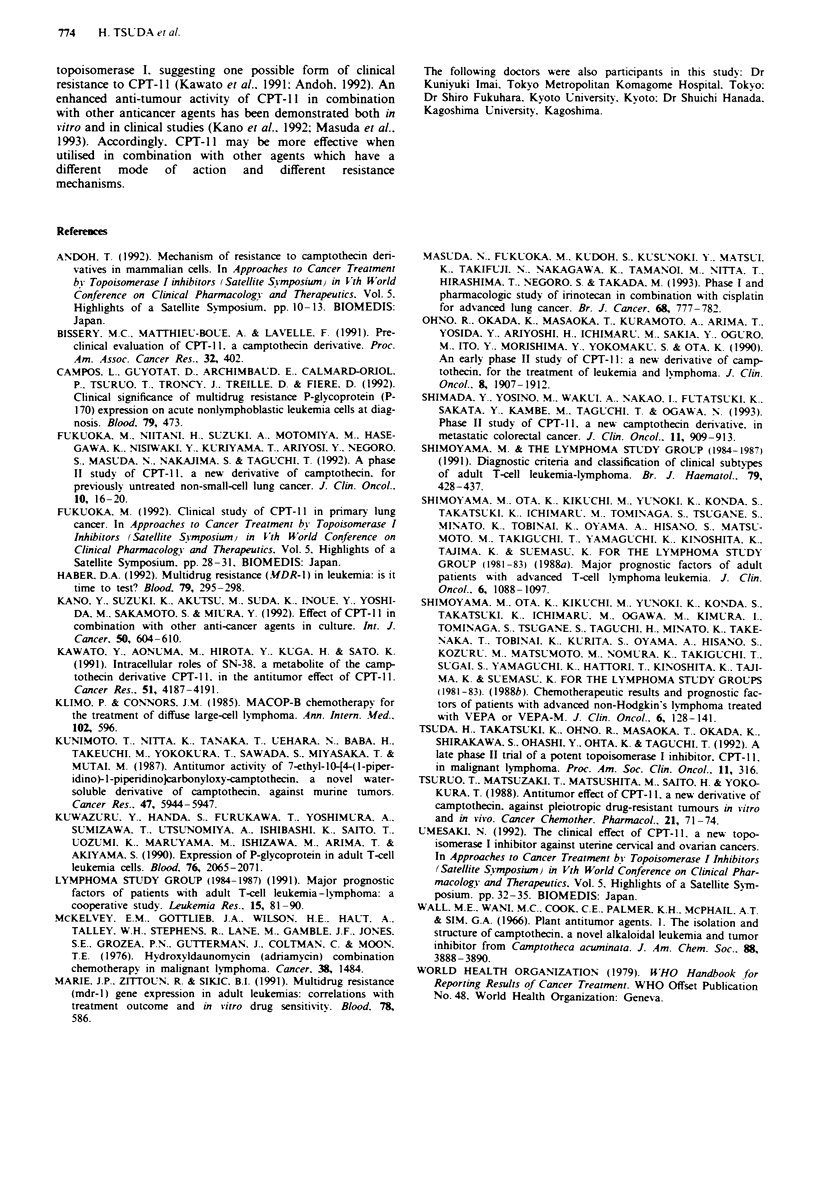# Treatment of adult T-cell leukaemia-lymphoma with irinotecan hydrochloride (CPT-11). CPT-11 Study Group on Hematological Malignancy.

**DOI:** 10.1038/bjc.1994.394

**Published:** 1994-10

**Authors:** H. Tsuda, K. Takatsuki, R. Ohno, T. Masaoka, K. Okada, S. Shirakawa, Y. Ohashi, K. Ota

**Affiliations:** Department of Internal Medicine, Kumamoto University Medical School, Japan.

## Abstract

A late phase II study of a new camptothecin analogue, irinotecan hydrochloride (CPT-11), was conducted to evaluate the anti-tumour effect and toxicity in patients with refractory leukaemia and lymphoma including adult T-cell leukaemia (ATL)-lymphoma, in a multi-institutional cooperative study. All the patients with ATL had been previously treated with various conventional combination chemotherapies and were refractory to these therapies or had relapsed. CPT-11 was administered at a dose of 40 mg m-2 day-1 for three consecutive days repeated weekly until evidence of disease progression. One complete remission and four partial remissions were achieved in 13 assessable patients with ATL. The median total dose to achieve remission was 240 mg m-2 and the median duration of response was 31 days. The major toxicities were leucopenia (83%), diarrhoea (62%) and nausea/vomiting (69%). These were relatively severe, but they were generally tolerable and reversible. However, one patient died probably as a result of this therapy. No effective chemotherapy for adult T-cell leukaemia-lymphoma has yet been established, and the prognosis for patients with this disease is very poor. Our results suggest that CPT-11 may be a promising agent for this disease. Further combination therapy with CPT-11 is needed to improve the therapy for ATL.


Br. J. Cancer (1994), 70, 771-774 © Macmillan Press Ltd., 1994

### Treatment of adult T-cell leukaemia-lymphoma with irinotecan hydrochloride (CPT-11)

H. Tsuda[1], K. Takatsuki[1], R. Ohno[2], T. Masaoka[3], K. Okada[4], S. Shirakawa[5], Y. Ohashi[6], K. Ota[7] & The CPT-11 Study Group on Hematological Malignancy, Tokyo, Japan

[1]*Department of Internal Medicine, Kumamoto University Medical School, 1-1-1 Honjo, Kumamoto 860, Japan;* [2]*Department of Internal Medicine, Hamamatsu University School of Medicine, 3600 Handacho, Hamamatsu 431-31, Japan;* [3]*Department of Internal Medicine, The Center for Adult Disease, 1-3-3 Nakamichi, Higashinari-ku, Osaka 537, Japan'* [4]*Department of Blood Transfusion, Hiroshima University School of Medicine, 1-2-3 Kasumi, Minami-ku, Hiroshima 734, Japan;* [5]*Department of Internal Medicine, Mie University School of Medicine, 2-174 Edobashi, Tsu 514, Japan;* [6]*School of Health Sciences and Nursing, Faculty of Medicine, University of Tokyo, 7-3-1 Hongo, Bunkyo-ku, Tokyo, Japan;* [7]*Nagoya Memorial Hospital, 4-305 Hirabari, Tenpaku-ku, Nagoya 468, Japan.*

**Summary** A late phase II study of a new camptothecin analogue, irinotecan hydrochloride (CPT-11), was conducted to evaluate the anti-tumour effect and toxicity in patients with refractory leukaemia and lymphoma including adult T-cell leukaemia (ATL)-lymphoma, in a multi-institutional cooperative study. All the patients with ATL had been previously treated with various conventional combination chemotherapies and were refractory to these therapies or had relapsed. CPT-11 was administered at a dose of 40 mg $m^{-2}$ $day^{-1}$ for three consecutive days repeated weekly until evidence of disease progression. One complete remission and four partial remissions were achieved in 13 assessable patients with ATL. The median total dose to achieve remission was 240 mg $m^{-2}$ and the median duration of response was 31 days. The major toxicities were leucopenia (83%), diarrhoea (62%) and nausea vomiting (69%). These were relatively severe, but they were generally tolerable and reversible. However, one patient died probably as a result of this therapy. No effective chemotherapy for adult T-cell leukaemia-lymphoma has yet been established, and the prognosis for patients with this disease is very poor. Our results suggest that CPT-11 may be a promising agent for this disease. Further combination therapy with CPT-11 is needed to improve the therapy for ATL.

The prognosis of adult T-cell leukaemia-lymphoma (ATL) is generally very poor (Shimoyama *et al.*, 1988*a*, 1991), and the median survival times (MSTs) for the acute, lymphoma and chronic types are 5.4, 10.2 and 24.3 months respectively (Shimoyama *et al.*, 1991). Chemotherapy for ATL has been largely unsuccessful because of tumour resistance, and it was recently reported that ATL cells frequently express the *MDR*-1 gene product, a membrane P-glycoprotein, at the time of presentation (Kuwazuru *et al.*, 1990). Thus, a new agent having a different mechanism of action is needed, and activity in models of multidrug resistance may be relevant.

Irinotecan hydrochloride (CPT-11) is a semisynthetic and water-soluble derivative of camptothecin, an anti-tumour alkaloid isolated from *Camptotheca acuminata* (Wall *et al.*, 1966). This compound has displayed excellent *in vitro* and *in vivo* anti-tumour activity in a variety of preclinical studies (Kunimoto *et al.*, 1987; Bissery *et al.*, 1991). CPT-11 acts through the inhibition of topoisomerase I (Kawato *et al.*, 1991; Andoh, 1992). CPT-11 is reported to be effective against vincristine- and doxorubicin-resistant P388 leukaemia both *in vitro* and *in vivo* (Tsuruo *et al.*, 1988), suggesting that it may be able to overcome multidrug resistance.

A late phase II study of CPT-11 was conducted in Japan to evaluate its anti-tumour effects and toxicity in patients with various types of leukaemia and lymphoma, including ATL (Tsuda *et al.*, 1992). In the present report, the results were analysed with the focus on ATL, in order to determine whether CPT-11 has any worthwhile activity against ATL.

### Patients and methods

### *Patients*

Patients were enrolled in this study if they fulfilled the following eligibility criteria: (1) histologically confirmed leukaemia or malignant lymphoma, (2) measurable and evaluable disease, (3) refractory to standard therapy, (4) no radiotherapy or chemotherapy within 2 weeks before entry, (5) life expectancy of at least 2 months, (6) performance status of 3 or better according to the Eastern Cooperative Oncology Group (ECOG), (7) adequate bone marrow, lung, liver, and renal function, (8) no serious complications, (9) no other active malignancies, (10) a negative reaction to skin test for CPT-11. Informed consent was obtained from all patients before entry. ATL was diagnosed and classified according to the criteria of the Lymphoma Study Group (Shimoyama *et al.*, 1991).

### *Study design*

This was an open, non-randomized phase II study in patients with refractory or relapsed leukaemia and lymphoma that was designed to evaluate the anti-tumour effect and toxicity of CPT-11. A complete blood count and biochemistry tests were routinely done. Response was judged by the Japan Society for Cancer Therapy in accordance with the criteria of the World Health Organization (World Health Organization, 1979). The eligibility, suitability and handling of each patient were determined by an evaluation committee. The study was approved by the ethics committee of each participating institution.

### *Treatment schedule*

Based on the results of an early phase II study, 40 mg $m^{-2}$ CPT-11 was administered as a 60 min intravenous infusion on three consecutive days per week repeated every week until disease progression or intolerable toxicity occurred. When myelosuppression and diarrhoea occurred, administration was postponed until their recovery.

### Results

### *Patients' background*

Patients with relapsed or refractory leukaemia and lymphoma were enrolled into this study from 34 Japanese institutions

Correspondence: H. Tsuda, Division of Clinical Haematology Immunology, Kumamoto City Hospital, Kotoh 1-1-60, Kumamoto 862, Japan.
Received 15 February 1994; and in revised form 3 June 1994.

**Table I** Clinical characteristics of the ATL patients

| *Patient no.* | *Sex* | *Age (years)* | *PS*[a] | *Type of ATL* | *Disease sites* | *No. of sites* | *LDH*[b] *(IU $l^{-1}$)* | *Ca*[c] *(mg $dl^{-1}$)* | *No. of previous agents* | *Disease status* |
|---|---|---|---|---|---|---|---|---|---|---|
| 2 | M | 63 | 2 | Lymphoma | Abdominal LN[d] tonsil | 2 | 1,413 | 10.8 | 8 | PrR[e] |
| 5 | M | 61 | 1 | Lymphoma | Mediastinal LN | 1 | 608 | 8.8 | 12 | PrR |
| 6 | M | 44 | 1 | Lymphoma | Superficial LN | 1 | 1,020 | 7.9 | 9 | RR[f] |
| 8 | F | 74 | 1 | Lymphoma | Superficial LN | 1 | 407 | 9.7 | 1 | PrR |
| 12 | M | 66 | 0 | Lymphoma | Superficial LN lung | 2 | 726 | 8.8 | 9 | R[g] |
| Mean | – | 61.8 | 1 | – | – | 1.2 | 835 | 9.2 | 7.8 | – |
| 1 | F | 70 | 3 | Lymphoma | Abdominal LN Tonsil | 2 | 2,943 | 13.4 | 6 | PrR |
| 3 | F | 78 | 2 | Acute | Peripheral blood Spleen | 2 | 822 | 10.6 | 7 | PrR |
| 4 | F | 67 | 3 | Lymphoma | Superficial LN | 1 | 935 | 8.7 | 3 | RR |
| 7 | M | 49 | 2 | Lymphoma | Superficial LN | 1 | 1,635 | 8.9 | 6 | R |
| 9 | F | 60 | 3 | Acute | Peripheral blood | 1 | 888 | 8.9 | 4 | PrR |
| 10 | M | 59 | 3 | Lymphoma | Abdominal LN Superficial LN Pleural fluid | 3 | 735 | 11.8 | 16 | RR |
| 11 | M | 72 | 3 | Acute | Peripheral blood Superficial LN Liver Spleen | 4 | 1,606 | 9.0 | 6 | PrR |
| 14 | M | 50 | 2 | Lymphoma | Superficial LN | 1 | 666 | 8.6 | 4 | RR |
| Mean | – | 63.1 | 2.6 | – | – | 1.9 | 1,279 | 10.0 | 6.5 | – |

[a]PS, performance status. [b]Normal range 200–450 IU $l^{-1}$. [c]Normal range 8.5–10.2 mg $dl^{-1}$. [d]LN, lymph node. [e]PrR, primary refractory. [f]R, relapsed. [g]RR, relapsed and refractory.

**Table II** Response and administration

| | *Patient no.* | *Response* | *Total dose (mg $m^{-2}$)* | *Time to CR/PR (days)* | *Dose to CR/PR (mg $m^{-2}$)* | *Duration of response (days)* |
|---|---|---|---|---|---|---|
| Responder | 2 | PR | 680 | 13 | 240 | 39 |
| | 5 | CR | 3,585 | 13 | 240 | 130 |
| | 6 | PR | 600 | 24 | 360 | 31 |
| | 8 | PR | 236 | 6 | 117 | 29 |
| | 12 | PR | 540 | 12 | 120 | 29 |
| | Median | – | 600 | 13 | 240 | 31 |
| Non-responder | 1 | NC | 330 | – | – | – |
| | 3 | PD | 240 | – | – | – |
| | 4 | NC | 480 | – | – | – |
| | 7 | PD | 760 | – | – | – |
| | 9 | NC | 500 | – | – | – |
| | 10 | PD | 410 | – | – | – |
| | 11 | PD | 160 | – | – | – |
| | 14 | PD | 360 | – | – | – |
| | Median | – | 385 | – | – | – |
| | Total median | – | 480 | – | – | – |

between February 1990 and March 1992. Seventy-nine patients were registered, including 14 patients with ATL from five institutions. Thirteen of 14 ATL patients were assessable for efficacy and toxicity (Table I). One patient (no. 13) was excluded from evaluation because of short interval of prior therapy before starting CPT-11. All patients had been treated previously with various conventional combination chemotherapies. Seven patients were refractory to the initial induction therapy, two had relapsed after achieving CR with the prior chemotherapy and four were relapsed and refractory to second or subsequent induction chemotherapy regimens.

*Response*

Among the 13 patients evaluated, one achieved CR and four achieved PR (Table II). The CR was obtained in patient no. 5, who had lymphoma type of ATL with massive mediastinal lymphadenopathy. The disease had been resistant to various prior forms of chemotherapy. CR was achieved after the second course of CPT-11, and it lasted for 130 days. All the PR patients also had lymphoma-type ATL and had received various forms of chemotherapy. PR was achieved at 13, 24, 6 and 12 days after the start of CPT-11 therapy and lasted for 39, 31, 29 and 29 days respectively. The median time to PR (including the CR patient) was 13 days and the median duration of PR was 31 days. The time course of the change in tumour size for each responder is outlined in Figure 1. A reduction in tumour size was apparent after only 1 week of treatment in four out of five patients. Complete disappearance of measurable lesions actually occurred in two patients (no. 5 and 12) after two and three courses of treatment respectively. However, patient no. 12 was not judged as having achieved CR because a radiologically visible but unmeasurable pulmonary deposit did not disappear completely after the treatment. The serum lactic acid dehydrogenase (LDH) levels of responders and non-responders

**Figure 1** The time course of the change in tumour size in CPT-11 responders. The size of tumours was measured as indicated by the Japan Society for Cancer Therapy in accordance with the criteria of the World Health Organization. Each point represents the tumour size relative to that just before CPT-11 therapy. □, Patient 2; ●, patient 5; ◁, patient 6; O, patient 8; ■, patient 12.

before treatment were $835 \pm 392$ and $1,279 \pm 770$ IU $l^{-1}$ (mean ± s.d.) respectively. The decrease in LDH levels of responders was 12–62% compared with pretreatment levels 2 weeks after the start of therapy (data not shown). The lower LDH level persisted for 8 weeks except in one patient whose LDH level remained moderately elevated.

*Toxicity*

The toxicities caused by CPT-11 therapy are listed in Table III. Myelosuppression was the most common toxicity observed: leucopenia was noted in 83%, anaemia in 67% and thrombocytopenia in 50%. Toxicity was generaly reversible, 2–3 weeks were required for recovery to the pretreatment level. Nausea and vomiting occurred in 69% and diarrhoea in 62%. All patients who experienced diarrhoea recovered. The median day of recovery from onset of diarrhoea was 4 days (range 1–34). Mild elevation of the level of glutamic oxaloacetic transaminase (GOT) and/or glutamic pyruvic transaminase (GPT) was seen in 15%. In addition, alopecia developed in 40%, but haemorrhagic cystitis was not observed. However, one patient (no. 4) died probably as a result of treatment for aspiration pneumonia caused by leucopenia.

### Discussion

The prognosis of adult patients with advanced peripheral T-cell lymphoma-leukaemia is generally poor, and ATL is the worst among this group of diseases (Shimoyama *et al.*, 1988*a*). Patients with ATL have been treated by various agents and schedules, such as VEPA (Shimoyama *et al.*, 1988*b*), CHOP (McKelvey *et al.*, 1976) and MACOP-B (Klimo *et al.*, 1985). In addition, treatment with etoposide, interferon-α and interferon-γ as single agents has been tried, but the survival benefit of any of these regimens remains uncertain (Shimoyama *et al.*, 1988*a*). The *MDR*-1 gene or P-glycoprotein seems to be involved in the development of drug resistance in various tumours including ATL (Marie *et al.*, 1991; Campos *et al.*, 1992; Haber, 1992). Thus, more effective agents and treatments for ATL are required.

The anti-tumour activity of CPT-11 is attributed to inhibition of topoisomerase I (Kawato *et al.*, 1991; Andoh, 1992). In addition, it has been shown that there is no cross-resistance between CPT-11 and adriamycin or vincristine both in animal tumour models and *in vitro* studies (Tsuruo *et al.*, 1988). Phase II trials in various tumour types, including lung (Fukuoka *et al.*, 1992*a*, *b*), ovary, cervical (Umesaki, 1992), colorectal cancer (Shimada *et al.*, 1993) and haematological malignancies (Ohno *et al.*, 1990), have been performed in Japan, and CPT-11 showed definite clinical response. The response rate of ATL patients in our study was 38% (5/13). The duration of response in our study was short, but our data do suggest a wide anti-tumour spectrum for CPT-11. This promising drug may be able to overcome multiple drug resistance related to the *MDR*-1 gene or P-glycoprotein in tumours such as ATL, but this at present remains speculative.

The major side-effects were myelosuppression and gastrointestinal toxicities. Leucopenia was more severe than thrombocytopenia, as was observed in previous clinical studies. Diarrhoea was also severe, but reversible. It could generally be minimised by a standard dose of an anticholinergic agent. Treatment sometimes had to be postponed owing to leucopenia and diarrhoea, but in general treatment was repeated as planned. However, one patient died as a result of this treatment. The patient experienced grade 3 leucopenia and diarrhoea and a deterioration in general condition which caused an aspiration pneumonia. Thus, in regard to these side-effects, special care must be taken and adequate supportive therapy is needed.

According to a recent large-scale study, five factors are associated with a reduced survival in ATL, including poor performance status, high LDH level, age >40 years, a greater number of lesions and hypercalcaemia (Lymphoma Study Group, 1991). All patients enrolled in our study received various previous forms of chemotherapy and had several adverse prognostic factors (Table I). This suggests that more satisfactory results may be achievable in untreated patients with better performance status.

In conclusion, our results show that CPT-11 is clinically effective against ATL. Some cell lines resistant to CPT-11 have recently been established and have altered forms of

**Table III** Major toxicities

| *Toxicity* | *No. of evaluable patients* | *Grade 1* | *2* | *3* | *4* | *%* |
|---|---|---|---|---|---|---|
| Leucopenia | 12 | 0 | 2 | 4 | 4 | 83 |
| Anaemia | 12 | 2 | 2 | 3 | 1 | 67 |
| Thrombocytopenia | 12 | 1 | 0 | 4 | 1 | 50 |
| Elevation of GOT GPT | 13 | 1 | 1 | 0 | 0 | 15 |
| Elevation of total bilirubin | 13 | 0 | 0 | 0 | 0 | 0 |
| Elevation of BUN | 13 | 0 | 0 | 0 | 0 | 0 |
| Diarrhoea | 13 | 1 | 1 | 6 | 0 | 62 |
| Nausea vomiting | 13 | 4 | 2 | 3 | – | 69 |
| Alopecia | 10 | 0 | 2 | 2 | – | 40 |

topoisomerase I, suggesting one possible form of clinical resistance to CPT-11 (Kawato et al., 1991; Andoh, 1992). An enhanced anti-tumour activity of CPT-11 in combination with other anticancer agents has been demonstrated both in vitro and in clinical studies (Kano et al., 1992; Masuda et al., 1993). Accordingly, CPT-11 may be more effective when utilised in combination with other agents which have a different mode of action and different resistance mechanisms.

The following doctors were also participants in this study: Dr Kuniyuki Imai, Tokyo Metropolitan Komagome Hospital, Tokyo; Dr Shiro Fukuhara, Kyoto University, Kyoto; Dr Shuichi Hanada, Kagoshima University, Kagoshima.

### References

ANDOH, T. (1992). Mechanism of resistance to camptothecin derivatives in mammalian cells. In *Approaches to Cancer Treatment by Topoisomerase I inhibitors (Satellite Symposium) in Vth World Conference on Clinical Pharmacology and Therapeutics*, Vol. 5, Highlights of a Satellite Symposium, pp. 10-13. BIOMEDIS: Japan.

BISSERY, M.C., MATTHIEU-BOUE, A. & LAVELLE, F. (1991). Preclinical evaluation of CPT-11, a camptothecin derivative. *Proc. Am. Assoc. Cancer Res.*, **32**, 402.

CAMPOS, L., GUYOTAT, D., ARCHIMBAUD, E., CALMARD-ORIOL, P., TSURUO, T., TRONCY, J., TREILLE, D. & FIERE, D. (1992). Clinical significance of multidrug resistance P-glycoprotein (P-170) expression on acute nonlymphoblastic leukemia cells at diagnosis. *Blood*, **79**, 473.

FUKUOKA, M., NIITANI, H., SUZUKI, A., MOTOMIYA, M., HASEGAWA, K., NISIWAKI, Y., KURIYAMA, T., ARIYOSI, Y., NEGORO, S., MASUDA, N., NAKAJIMA, S. & TAGUCHI, T. (1992). A phase II study of CPT-11, a new derivative of camptothecin, for previously untreated non-small-cell lung cancer. *J. Clin. Oncol.*, **10**, 16-20.

FUKUOKA, M. (1992). Clinical study of CPT-11 in primary lung cancer. In *Approaches to Cancer Treatment by Topoisomerase I Inhibitors (Satellite Symposium) in Vth World Conference on Clinical Pharmacology and Therapeutics*, Vol. 5, Highlights of a Satellite Symposium, pp. 28-31, BIOMEDIS: Japan.

HABER, D.A. (1992). Multidrug resistance (*MDR*-1) in leukemia: is it time to test? *Blood*, **79**, 295-298.

KANO, Y., SUZUKI, K., AKUTSU, M., SUDA, K., INOUE, Y., YOSHIDA, M., SAKAMOTO, S. & MIURA, Y. (1992). Effect of CPT-11 in combination with other anti-cancer agents in culture. *Int. J. Cancer*, **50**, 604-610.

KAWATO, Y., AONUMA, M., HIROTA, Y., KUGA, H. & SATO, K. (1991). Intracellular roles of SN-38, a metabolite of the camptothecin derivative CPT-11, in the antitumor effect of CPT-11. *Cancer Res.*, **51**, 4187-4191.

KLIMO, P. & CONNORS, J.M. (1985). MACOP-B chemotherapy for the treatment of diffuse large-cell lymphoma. *Ann. Intern. Med.*, **102**, 596.

KUNIMOTO, T., NITTA, K., TANAKA, T., UEHARA, N., BABA, H., TAKEUCHI, M., YOKOKURA, T., SAWADA, S., MIYASAKA, T. & MUTAI, M. (1987). Antitumor activity of 7-ethyl-10-[4-(1-piperidino)-1-piperidino]carbonyloxy-camptothecin, a novel water-soluble derivative of camptothecin, against murine tumors. *Cancer Res.*, **47**, 5944-5947.

KUWAZURU, Y., HANDA, S., FURUKAWA, T., YOSHIMURA, A., SUMIZAWA, T., UTSUNOMIYA, A., ISHIBASHI, K., SAITO, T., UOZUMI, K., MARUYAMA, M., ISHIZAWA, M., ARIMA, T. & AKIYAMA, S. (1990). Expression of P-glycoprotein in adult T-cell leukemia cells. *Blood*, **76**, 2065-2071.

LYMPHOMA STUDY GROUP (1984-1987) (1991). Major prognostic factors of patients with adult T-cell leukemia-lymphoma: a cooperative study. *Leukemia Res.*, **15**, 81-90.

MCKELVEY, E.M., GOTTLIEB, J.A., WILSON, H.E., HAUT, A., TALLEY, W.H., STEPHENS, R., LANE, M., GAMBLE, J.F., JONES, S.E., GROZEA, P.N., GUTTERMAN, J., COLTMAN, C. & MOON, T.E. (1976). Hydroxyldaunomycin (adriamycin) combination chemotherapy in malignant lymphoma. *Cancer*, **38**, 1484.

MARIE, J.P., ZITTOUN, R. & SIKIC, B.I. (1991). Multidrug resistance (mdr-1) gene expression in adult leukemias: correlations with treatment outcome and *in vitro* drug sensitivity. *Blood*, **78**, 586.

MASUDA, N., FUKUOKA, M., KUDOH, S., KUSUNOKI, Y., MATSUI, K., TAKIFUJI, N., NAKAGAWA, K., TAMANOI, M., NITTA, T., HIRASHIMA, T., NEGORO, S. & TAKADA, M. (1993). Phase I and pharmacologic study of irinotecan in combination with cisplatin for advanced lung cancer. *Br. J. Cancer*, **68**, 777-782.

OHNO, R., OKADA, K., MASAOKA, T., KURAMOTO, A., ARIMA, T., YOSIDA, Y., ARIYOSHI, H., ICHIMARU, M., SAKIA, Y., OGURO, M., ITO, Y., MORISHIMA, Y., YOKOMAKU, S. & OTA, K. (1990). An early phase II study of CPT-11: a new derivative of camptothecin, for the treatment of leukemia and lymphoma. *J. Clin. Oncol.*, **8**, 1907-1912.

SHIMADA, Y., YOSINO, M., WAKUI, A., NAKAO, I., FUTATSUKI, K., SAKATA, Y., KAMBE, M., TAGUCHI, T. & OGAWA, N. (1993). Phase II study of CPT-11, a new camptothecin derivative, in metastatic colorectal cancer. *J. Clin. Oncol.*, **11**, 909-913.

SHIMOYAMA, M. & THE LYMPHOMA STUDY GROUP (1984-1987) (1991). Diagnostic criteria and classification of clinical subtypes of adult T-cell leukemia-lymphoma. *Br. J. Haematol.*, **79**, 428-437.

SHIMOYAMA, M., OTA, K., KIKUCHI, M., YUNOKI, K., KONDA, S., TAKATSUKI, K., ICHIMARU, M., TOMINAGA, S., TSUGANE, S., MINATO, K., TOBINAI, K., OYAMA, A., HISANO, S., MATSUMOTO, M., TAKIGUCHI, T., YAMAGUCHI, K., KINOSHITA, K., TAJIMA, K. & SUEMASU, K. FOR THE LYMPHOMA STUDY GROUP (1981-83) (1988*a*). Major prognostic factors of adult patients with advanced T-cell lymphoma leukemia. *J. Clin. Oncol.*, **6**, 1088-1097.

SHIMOYAMA, M., OTA, K., KIKUCHI, M., YUNOKI, K., KONDA, S., TAKATSUKI, K., ICHIMARU, M., OGAWA, M., KIMURA, I., TOMINAGA, S., TSUGANE, S., TAGUCHI, H., MINATO, K., TAKENAKA, T., TOBINAI, K., KURITA, S., OYAMA, A., HISANO, S., KOZURU, M., MATSUMOTO, M., NOMURA, K., TAKIGUCHI, T., SUGAI, S., YAMAGUCHI, K., HATTORI, T., KINOSHITA, K., TAJIMA, K. & SUEMASU, K. FOR THE LYMPHOMA STUDY GROUPS (1981-83). (1988*b*). Chemotherapeutic results and prognostic factors of patients with advanced non-Hodgkin's lymphoma treated with VEPA or VEPA-M. *J. Clin. Oncol.*, **6**, 128-141.

TSUDA, H., TAKATSUKI, K., OHNO, R., MASAOKA, T., OKADA, K., SHIRAKAWA, S., OHASHI, Y., OHTA, K. & TAGUCHI, T. (1992). A late phase II trial of a potent topoisomerase I inhibitor, CPT-11, in malignant lymphoma. *Proc. Am. Soc. Clin. Oncol.*, **11**, 316.

TSURUO, T., MATSUZAKI, T., MATSUSHITA, M., SAITO, H. & YOKOKURA, T. (1988). Antitumor effect of CPT-11, a new derivative of camptothecin, against pleiotropic drug-resistant tumours *in vitro* and *in vivo*. *Cancer Chemother. Pharmacol.*, **21**, 71-74.

UMESAKI, N. (1992). The clinical effect of CPT-11, a new topoisomerase I inhibitor against uterine cervical and ovarian cancers. In *Approaches to Cancer Treatment by Topoisomerase I Inhibitors (Satellite Symposium) in Vth World Conference on Clinical Pharmacology and Therapeutics*, Vol. 5, Highlights of a Satellite Symposium, pp. 32-35. BIOMEDIS: Japan.

WALL, M.E., WANI, M.C., COOK, C.E., PALMER, K.H., McPHAIL, A.T. & SIM, G.A. (1966). Plant antitumor agents. 1. The isolation and structure of camptothecin, a novel alkaloidal leukemia and tumor inhibitor from *Camptotheca acuminata*. *J. Am. Chem. Soc.*, **88**, 3888-3890.

WORLD HEALTH ORGANIZATION (1979). *WHO Handbook for Reporting Results of Cancer Treatment*. WHO Offset Publication No. 48, World Health Organization: Geneva.